# The Role of Vagus Nerve Stimulation in Depression: What We Know?

**DOI:** 10.1192/j.eurpsy.2022.1449

**Published:** 2022-09-01

**Authors:** A. Fraga, B. Mesquita, D. Esteves-Sousa, J. Facucho-Oliveira, M. Albuquerque, P. Espada-Santos, P. Cintra, A. Moutinho

**Affiliations:** Hospital de Cascais, Psychiatry, Alcabideche, Portugal

**Keywords:** Depression, Treatment-resistant depression, vagus nerve stimulation

## Abstract

**Introduction:**

Depression is a leading cause of disability affecting over 300 million indivuals worldwide. About 1/3 of patients with depression fail to achive remission despite treatment with multiple antidepressants and are considered to have treatment-resistant depression (TRD). In view of such facts, vagus nerve stimulation (VNS) therapy was approved as an adjunctive long-term treatment for TRD.

**Objectives:**

The authors elaborate a narrative literature review about de effectiveness of VNS in treatment for TRD.

**Methods:**

PubMed database searched using the terms “treatment-resistant depression”, “vagus nerve stimulation”

**Results:**

The pathophysiology of depression is complex and includes social environmental stress factors, genetic and biological processes, inflammation, and disturbances in monoamine neurotransmission. The overdrive of the HPA axis is most consistently seen in subjects with more severe depression, when the cortisol feedback inhibitory mechanisms are impaired, contributing to cytokine oversecretion. It has been shown that chronic exposure to elevated inflammatory cytokines can lead to depression. The vagus nerve represents the main component of the parasympathetic nervous system, which oversees a vast array of crucial bodily functions, including control of mood and imune response. VNS therapy has a demonstrated anti-inflammatory effect which might be a significant reason for its efficacy in patients who did not respond to antidepressants. Treatments thar target the vagus nerve increase the vagal tone and inhibit cytokine production and the stimulation of vagal aferente fibers in the gut influences monaminergic brain systems.

**Conclusions:**

The mecanismos by which VNS may benefit patients nonresponsive to conventional antidepressants is unclear, with further research need to clarify this.

**Disclosure:**

No significant relationships.

